# 

**Published:** 1893-05

**Authors:** 


### PAMPHLETS RECEIVED.

Skin-Grafting upon the Cranium. By F. C. Schaefer, M. D., Chicago.

Vertebral Surgery, with Report of Three Cases. By F. C. Schaefer, M. D., Chicago.

Contributions to the Study of Cerebro-Spinal Meningitis. By Wm. B. Pritchard, M. D., New York.

Methods of Precision in the Investigation of Disorders of Digestion. By J. H. Kellog, M. D., Battle Creek, Michigan.

Habitual Abortion. By E. S. McKee, M. D., Cincinnati. Reprint.

Colpo-Perineorraphy. By Edward W. Jenks, M. D., Detroit. Reprint.

Homeopathy and its Congeners. By G. Frank Lydston, M. D., Chicago.

Abscess Around the Rectum. By Chas. B. Kelsey, M. D., New York.

Cosmetic Surgery of the Nose. By John B. Roberts, M. D., Philadelphia.

Wintering in Egypt. By Frederick Peterson, M. D., New York. Reprint.

Hystero-Epilepsy, with Report of Cases. By Dr. A. Vander Veer, Albany, N. V.

New York Letters on Orthopedic Surgery. By Dr. Stuart McCurdy, Dennison, Ohio.

Excision of Tubercular Knee-Joint. By H. Augustus Wilson, M. D., Philadelphia. Reprint.

Quarantine Control. State or National? A speech by Joseph Holt, M. D., New Orleans, La.

Uterine Hemorrhage, Puerperal and Non-Puerperal. By Dr. A. Vander Veer, Albany, N. Y.

Bloodless Amputation at the Hip-Joint by a New Method. By Nicholas Senn, M. D., Chicago.

Gastrostomy in Carcinoma of the Cardiac Orifice. By Dr. Emory Lanphear, Kansas City, Mo.

Arterial Saline Infusion, with Report of Cases. By Robert H. M. Dawbarn, M. D., New York.

Some Contributions to the Study of the Muscular Sense. By Geo. J. Preston, M. D., Baltimore. Reprint.

The Collegiate Degree as an Evidence of Fitness for the Study of Medicine. By Dr. L. Harrison Mettler.

The Management of Cancer of the Uterus, Complicated by Pregnancy. By Dr. A. Vander Veer, Albany, N. Y.

Typhoid Fever in the Light of Modern Research. Facts and Doubts about Cholera. By Dr. L. Bremer, St. Louis.

Intercranial Neurectomy of Second and Third Divisions of Fifth Nerve. By John B. Roberts, M. D., Philadelphia.

Early Symptoms of Hip Disease and Etiology. Treatment of Abscess in Hip Disease. By H. Augustus Wilson, M. D., Philadelphia. Reprint.

